# Two Unrelated Iranian Patients with Adenosine Deaminase 2 Deficiency: A Case Report and Review of Treatment

**DOI:** 10.1155/2024/4380689

**Published:** 2024-08-12

**Authors:** Parvaneh Karimzade, Aziz Eghbali, Mohammad Keramatipour, Reza Shiari, Zahra Golchehre, Mahdieh Taghizadeh, Mazdak Fallahi, Shahrzad Fallah, Nasrin Khakbazan Fard, Narges Eslami, Narges Bazgir, Mahnaz Jamee, Zahra Chavoshzadeh

**Affiliations:** ^1^ Pediatric Neurology Research Center Research Institute for Children's Health Shahid Beheshti University of Medical Sciences, Tehran, Iran; ^2^ Department of Pediatrics School of Medicine Iran University of Medical Sciences, Tehran, Iran; ^3^ Department of Medical Genetics School of Medicine Tehran University of Medical Sciences, Tehran, Iran; ^4^ Division of Pediatric Rheumatology Department of Pediatrics Mofid Children's Hospital Shahid Beheshti University of Medical Sciences, Tehran, Iran; ^5^ Watson Genetic Laboratory, North Kargar Street, Tehran, Iran; ^6^ Immunology and Allergy Department Mofid Children's Hospital Shahid Beheshti University of Medical Sciences, Tehran, Iran; ^7^ Hearing Disorders Research Center Loghman Hakim Hospital Shahid Beheshti University of Medical Sciences, Tehran, Iran

## Abstract

**Background:**

Adenosine deaminase deficiency 2 (DADA2) is an autoinflammatory disorder, caused by the *CECR1* gene mutation. The major clinical manifestations include recurrent vasculitis, neurological disorders such as stroke, hematologic abnormalities, and immunodeficiency. As reported in previous studies, DADA2 may be manifested by ischemic or hemorrhagic strokes. This disorder also includes various hematological manifestations (pure red cell aplasia, pancytopenia, hemolytic anemia, and pancytopenia with bone marrow involvement). *Case Presentation*. In this case report, we present the clinical and immunological findings of two unrelated patients with DADA2. The first patient was a 7-year-old female who experienced recurrent neurological symptoms such as vertigo, tinnitus, hearing loss, and right-sided hemiparesis. Her brain magnetic resonance imaging (MRI) revealed a left-sided stroke, and she responded well to antitumor necrosis factor alpha agents and plasmapheresis. The second patient was a 6-year-old female who had recurrent fever and bicytopenia, aphthous lesions, cervical lymphadenopathy, and elevated liver enzymes. We also discussed the strategies used to manage the clinical manifestations in these two DADA2 patients.

**Conclusion:**

In this case report, we discussed two cases with DADA2 deficiency and their respective manifestations. The first case showed neurological symptoms while the second case had hematological symptoms. Although there is no established treatment for DADA2 due to its rarity, steroids are commonly used to treat this disorder. Antitumor necrosis factor is also effective in controlling the symptoms, especially the neurological ones. In cases where there is no appropriate response to these treatments, hematopoietic stem cell transplantation can be beneficial.

## 1. Introduction

The adenosine deaminase 2 deficiency (DADA2) is a monogenic inborn error of immunity with predominant autoinflammatory manifestations. This disorder was first described in 2014 in patients with fever, polyarteritis nodosa (PAN), livedo reticularis, liver disease, and a wide range of neurological manifestations [1, 2]. The adenosine deaminase 2 (ADA2) is a product of cat eye syndrome chromosome region 1 gene (*CECR1*) which is located on chromosome 22q11. Defects of this enzyme result in disturbances in the preventive mechanisms of endothelial cell damage. It is currently unknown what the exact prevalence of DADA2 deficiency is in Iran. The manifestations of DADA2 are widely diverse depending on the affected organ and the size of the involved arteries [3]. ADA2 deficiency has three main types such as hematological, immunodeficient, and vasculitis predominant types [4]. Neurological involvement is one of the most frequent manifestations in DADA2 patients with a rate of 50%–77%. These types of manifestations include transient ischemic attack, ischemic and hemorrhagic strokes, and a wide range of cranial neuropathies, with different intensities [5, 6, 7, 8, 9]. Hematological symptoms of DADA2 include pure red cell aplasia, neutropenia, thrombocytopenia, hemolytic anemia, and pancytopenia. Diagnosis is confirmed by a genetic test or determination of ADA2 activity levels [9, 10, 11, 12, 13, 14]. The early diagnosis and treatment of DADA2 are essential to prevent further possible lethal complications. Considering the different phenotypes of this disorder, its management policies could be distinctive [10]. In this paper, we present two cases of DADA2, one with neurological manifestations and the other with bicytopenia, along with their management.

## 2. Case Presentation

### 2.1. Case 1

A 7-year-old female patient has been experiencing recurring neurological symptoms for 1.5 years. She was born full-term to consanguineous parents (first cousins). The family history was noncontributory, and her only 4-year-old sibling was healthy. Neurodevelopmental milestones were within normal limits before the initiation of the neurological problems.

The first episode presented as ataxia, multiple cranial neuropathies manifested as ophthalmoplegia and hoarseness following an upper respiratory tract infection. The patient underwent brain magnetic resonance imaging (MRI) which yielded normal results. She was treated for suspected vasculitis with corticosteroids. The ophthalmoplegia resolved within a day, but the ataxia persisted.

**Figure 1:**
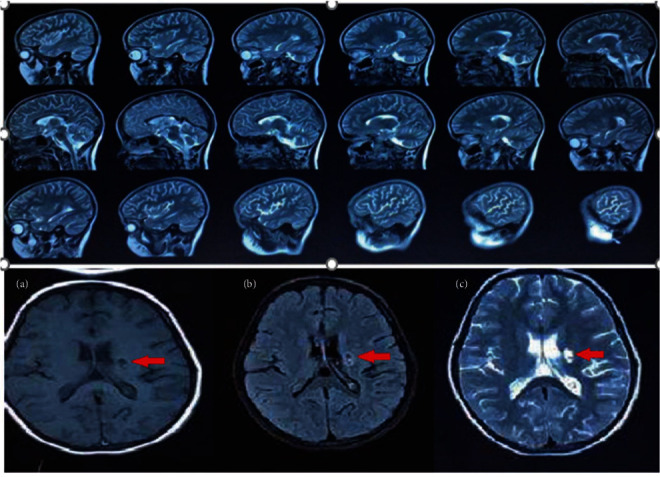
Sagittal T-2 weighted MRI images show mild cerebellar atrophy and brainstem involvement. Axial T-1 weighted (a), gad enhanced (b), and T-2 weighted (c) images of brain MRI demonstrate a focus of left internal capsule infarction (red arrows).

The second episode occurred 1 month later with peripheral facial nerve palsy. Further clinical details are lacking, but the palsy improved over time following a few physiotherapy sessions. Several months later, the patient developed acute vertigo, tinnitus due to vestibulocochlear nerve involvement, and right-sided hemiparesis. Brain MRI findings were suggestive of left-sided brain stroke (left internal capsule; Figure 1).

She was treated with enoxaparin, methylprednisolone, and intravenous immunoglobulin (IVIG) and discharged after few days. One week after discharge, she developed an unexplained high-grade fever together with an elevated erythrocyte sedimentation rate (ESR) and C-reactive protein (CRP). She was treated with intravenous antibiotics for 2 weeks.

She was hospitalized again 2 months later with complaints of vertigo, bilateral temporal headache, unilateral hearing loss, and severe tinnitus. Auditory brainstem response (ABR) showed moderate to severe hearing loss in the left ear. Cotrimoxazole, biotin, L-carnitine, and vitamin E were prescribed throughout treatment.

Finally, in the last episode, she presented with a decreased level of consciousness, severe vertigo, and deafness. At that time, ABR demonstrated mild to moderate hearing loss in the right ear and profound hearing loss in the left ear. After treatment with methylprednisolone, the patient became alert and had no cognitive impairment but was unable to communicate verbally due to the hearing loss. Ataxia, mild right hemiparesis, and facial palsy recurred, and the patient developed dysarthria and livedo reticularis on her limbs. Physical examinations revealed hypertonia and three plus deep tendon reflexes in all her extremities. Brain MRI revealed mild cerebellar atrophy and brainstem involvement. No difference was observed between the two MRI (Figure 1). Thyroid function tests, ophthalmologic exams, abdominal ultrasonography, EEG, thoracic and lumbosacral spine MRI, and nerve conduction velocity tests were all normal. Echocardiography revealed mild tricuspid regurgitation and a mild mitral regurgitation.

Laboratory findings included increased inflammatory markers and a complement factor C3 of 187 mg/dL, while lupus anticoagulant, other anticoagulant and procoagulant factors, anticardiolipin IgG and IgM, anti-beta-2 glycoprotein, C4, and CH50 were all within the normal limits. All immunoglobulin types were lower than normal range except for IgE. Furthermore, the number of lymphocytes CD3 and CD8 were also decreased (Table 1).

Finally, a whole-exome sequencing (WES) study revealed a reported homozygous missense variant in exon 2 of the ADA2 gene (NM_001282225) and c.140G>T (p.G47V). Based on ACMG guidelines [14], this variant can be classified as a pathogenic variant.

**Figure 2:**
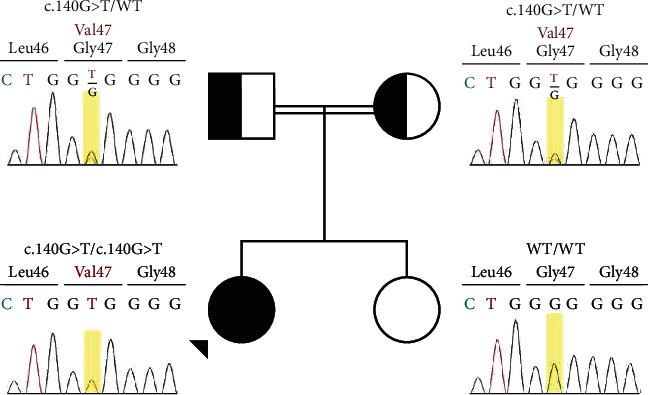
Homozygosity of the case 1 and heterozygosity of her parents for c.140G>T variant.

The ADA2 variant investigation was also performed for the parents, which disclosed the presence of same heterozygous variant in both parents. The patient's healthy sister was normal homozygote (Figure 2).

When the diagnosis of DADA2 was confirmed, treatment with an antitumor necrosis factor alpha (anti-TNF*α*) agent (infliximab) and plasmapheresis were recommended. Besides, an evaluation of the sibling and a discussion of probable future pregnancies was performed. The patient responded well to the administered treatments. No further neurological manifestations occurred. She is currently under investigation for allogeneic bone marrow transplantation from her HLA-identical sibling donor.

### 2.2. Case 2

A 6-year-old girl from consanguineous parents (third cousins) was admitted to the hospital due to intermittent fever since a month ago, aphthous lesion in mouth, weakness, cervical lymphadenopathy, and bicytopenia with an admission diagnosis of typhoid fever. The bicytopenia existed a month before the typhoid fever. She has two other siblings with a history of lymphadenopathy and elevated liver enzymes due to Epstein–Barr virus (EBV) infection.

In her laboratory data, lymphopenia was evident. In addition, her bone marrow aspiration was markedly hypocellular with infiltration and focal aggregation of lymphoid cells.

Liver function tests, lactate dehydrogenase (LDH), ferritin, and CRP were all elevated. Albumin was slightly decremented. Autoantibodies including anti-Sjogren's-syndrome-related antigen A (anti-SSA), anti-Sjogren's-syndrome type B (anti-SSB), fluorescent antinuclear antibody (FANA), and anti-double-stranded DNA antibody (anti-dsDNA) were negative, indicating the absence of autoimmune diseases. Hyper IgG and IgA were also observed. NBT test and dihydrorhodamine (DHR) were normal, while lymphocyte transformation tests were lower than normal. All immunologic tests are illustrated in detail in Table 1. Moreover, double-negative T cell was negative. Infectious laboratory workups revealed a positive Widal test. Salmonella Paratyphi C was detected in the stool culture test. All CMV, EBV, HIV, and parvovirus B19 were negative.

Computed tomography (CT) scan of the thorax demonstrated mild enlarged lymph in both axillae. Furthermore, a CT scan of the abdomen and pelvis indicated mild hepatosplenomegaly. She received treatment with broad-spectrum antibiotics, caspofungin, and G-CSF, but did not show significant improvement. An excisional biopsy of cervical lymph nodes was performed. Morphologic study and immunohistochemistry staining of cervical lymph nodes were consistent with reactive lymphadenitis. According to a family history of EBV infection in her siblings, bicytopenia, cervical lymphadenopathy, elevated liver enzyme, and exclusion of malignancy by hematologists, an underlying immunodeficiency was highly suspected, and immunologic laboratory tests were performed (Table 1).

Table 1 illustrates the details of laboratory work-ups in two presented cases.

As shown in Table 1, the patient exhibited low levels of lymphocytes and neutrophils, a low CD3 and CD4 count, and an abnormal level of immunoglobulins and lymphocyte transformation test. Taken together, these abnormal test results strongly suggest an immunodeficiency diagnosis.

Meanwhile, she received corticosteroid therapy, but her clinical manifestations did not improve (before administrating corticosteroids, all suspected infections such as EBV and CMV were ruled out). After 2 months, she was readmitted with fever and diarrhea. Unfortunately, she was expired due to septic shock while her chest CT scan was in favor of COVID-19. The requested WES before her death showed a pathogenic nonsense variant (c.934C>T; p.R312^*∗*^) in ADA2 gene. The family refused to undergo segregation analysis.

## 3. Discussion

The DADA2 is a monogenic inborn error of immunity usually responsible for the stroke and systemic vasculitis in pediatric population [1, 2, 12]. ADA2 is an enzyme that has a role in endothelial stability [1, 2]. Therefore, DADA2 may predispose the patient to vascular diseases, frequently in small- and medium-sized arteries [15]. Lack of the *CECR1* gene expression also shifts myeloid cells into proinflammatory cells, which increases inflammatory cytokines and results in tissue damage [1].

One of the most frequent clinical features of DADA2 is early-onset recurrent strokes, especially in patients with PAN-like manifestations [2, 16]. The majority of the reported strokes caused by ADA2 deficiency are ischemic, while many hemorrhagic strokes were also described [2, 17, 18]. Zhou et al. [2] were the first to report on the phenotypic and genotypic characteristics of nine cases of DADA2. Out of these nine cases, five of them had experienced early-onset stroke before the age of five, which was accompanied by inflammatory symptoms in each episode, with or without fever. MRI findings demonstrated lacunar stroke as acute or chronic small subcortical infarcts involving the brainstem, and the deep-brain nuclei [2]. The second episode in the first case consisted of focal neurological findings due to the occurrence of an ischemic stroke, and imaging studies were also evident for lacunar strokes.

Ischemic infarcts caused by DADA2 usually involve the small and deep-brain structures. In our case, the brainstem was affected. Since small vessels are involved in ADA2, infarctions may not manifest in MRI [15, 19].

The reported ataxia, dysarthria, hoarseness, and cognitive impairment could be due to recurrent and accumulative ischemic strokes [3].

Hearing involvement is a very rare manifestation of DADA2 [20]. A case of DADA2 deficiency with hearing involvement was reported by Westendorp et al. [21]. On the 11th birthday of the reported case, the patient was admitted to the hospital with nausea, vertigo, right-sided hearing loss, and nystagmus. His symptoms were interpreted as labyrinthitis [21]. Both of the auditory nerves were affected in our case, which is not as typical. In a multicenteric study by Caorsi et al. [22], three out of 20 patients had unilateral sensorineural hearing loss. There are also several reports on hearing deficits in patients with ADA deficiency [23]. The exact mechanism of hearing deficient in ADA deficiency is not fully determined. The infections, drugs, autoimmunity, and hypoxia are among the probable [24]. All the proposed causes are nearly in common between ADA deficiency and DADA2.

Ophthalmological involvements vary based on underlying cerebrovascular diseases. In 2019, Aksentijevich et al. [15] provided a detailed description of a disease and categorized its clinical findings into four groups: systemic autoinflammation, vasculitis, dysregulation of immune function, and hematologic abnormalities. They also stated that the accumulation of the effects of small and initially unrecognized strokes can lead to severe neurological deterioration over time, resulting in persistent dysarthria, ataxia, cranial nerve palsies, and cognitive dysfunction. Most of these manifestations were observed in our first patient.

In addition to the neurological manifestations of the first case, she also experienced livedo reticularis. The dominant vasculitis features are skin manifestations, mainly in form of livedo racemosa/reticularis [5, 12, 25, 26]. In a study conducted by Ashari et al. [27], 11 DADA2 patients were evaluated. All the studied patients had livedo racemosa/reticularis. Only one of the presented cases manifested livedo reticularis.

Recently, hematological symptoms associated with DADA2 have been identified as closely related to immunological symptoms. Pure red cell aplasia, autoimmune neutropenia, thrombocytopenia, hemolytic anemia, and pancytopenia are all included in hematological symptoms [17, 18, 19]. In the second case that was discussed earlier, it was clear that the patient was suffering from bicytopenia. Previously, a bicytopenic ADA2 deficient patient was reported with myelofibrosis [5]. In the second case, hypocellular marrow with infiltration and focal aggregation of lymphoid cells were observed in her bone marrow aspiration. In a study of five DADA2 patients, two patients had pure red cell aplasia, and their laboratory findings and clinical manifestations were consistent with Diamond–Blackfan anemia. One other manifested hemolytic anemia with hypercellular bone marrow [6]. ADA2 also has an essential role in B cell; therefore, cytopenia's predominant phenotype is closely related to immunodeficiency type [2, 28, 29].

It seems that the early diagnosis of DADA2 is of high importance. Based on the clinical variety and lack of comprehensive data, there is no established standard treatment yet. Steroids used to be the primary treatment of DADA2 in the past, with variable degrees of success and recurrence of the symptoms after tapering or discontinuation [1, 2, 20], as seen in the first presented patient as well. Other immunosuppressive treatments, such as azathioprine, cyclosporine, and cyclophosphamide, have shown little to no success in treating severe cases [1, 2, 20]. Carosi et al. [22] mentioned thalidomide and anti-TNF agents as the most effective drugs, with thalidomide achieving a complete response in six patients. Recently, it has been proposed that the lack of ADA2 could trigger the neutrophils and macrophages to produce a higher amount of TNF-*α* [30]. This hypothesis may explain the efficacy of TNF-*α* inhibitors in managing the symptoms of the disease. We were able to effectively treat the neurological symptoms in our case by using TNF-*α* inhibitors. In contrast, TNF-*α* inhibitors are less effective in patients with hematological and immunological phenotypes. These patients may benefit more from hematopoietic stem cell transplantation (HSCT). All patients who underwent HSCT have experienced remission of hematological, immunological, and even vasculitis manifestations. The ADA2 level and key cytokines such as TNF-*α*, interferon (INF)-*α*, and interleukin 6 were normalized after transplantation [18, 31, 32, 33].

In 2015, Van Eyck et al. [18] first reported a successfully performed allogeneic HSCT from a healthy sibling in a 6-month-old patient which resulted in a disease-free period of 5 years without using any medication. However, autoimmunity and graft-versus-host disease are potential complications of HSCT that should be taken into consideration. In 2017, Hashem et al. [17] reported on 14 DADA2 patients from six countries who underwent HSCT with a median age of 7.5 years. After a median follow-up period of 18 months, all patients remained asymptomatic and were clinically stable. The authors concluded that HSCT is a practical and definitive treatment option for patients with DADA2.

Recombinant manufacturing ADA2 enzyme is one of the hypothetical solutions. Since monocytes and macrophages, the major producers of ADA2, exist in bone marrow, bone marrow transplantation or genetic manipulation of marrow cells can be an applicable treatment option for these patients [2].

Another therapeutic option is fresh frozen plasma (FFP) infusion. Sufficient amounts of ADA2 are present in plasma; thus FFP infusion could be considered as a substitute for ADA2 activity. Sahin et al. [7] reported the initially successful treatment of DADA2 by monthly infusion of FFP. This strategy is limited by the short half-life of ADA2, large volumes of FFP, patients' physiologic incongruity, and severe disease episodes after cessation [1, 2, 5, 33].

## 4. Conclusion

In this report, we have delved into two unique cases of DADA2 deficiency and the specific symptoms that were observed in each case. The first case presented neurological symptoms, such as vertigo and tinnitus, while the second case exhibited hematological symptoms, like bicytopenia. Despite the rarity of this disorder, steroids remain the most commonly prescribed treatment for DADA2. Additionally, antitumor necrosis factor therapy can be effective in managing symptoms, particularly those related to the nervous system. Last, individuals who experience inappropriate immune responses may benefit from HSCT, which involves replacing the damaged bone marrow with healthy stem cells from a donor.

## Figures and Tables

**Table 1:** Laboratory investigations.

Laboratory parameters	Patient 1 (age: 7 years)	Patient 2 (age: 6 years)	Reference value
WBC (cells in mm^3^)	9,500	1,400	4,500–13,500
Lymphocyte (cells in mm^3^)	3,372.5	**1,106**	1,500–7,000
Neutrophil (cells in mm^3^)	4,902	**252**	1,000–8,000
IgG (mg/dL)	**453**	**5,005**	462–1,682
IgM (mg/dL)	**18**	192	38–251
IgA (mg/dL)	**19**	**782**	34–274
IgE (IU/mL)	1.4	8.93	<68
Anti-D IgG (IU/mL)	**0.13**	**0.46**	<0.1: no response 0.1−1: poor response >1: normal response
Anti-T IgG (IU/mL)	**0.2**	**2.6**	<0.1: no response 0.1−1: poor response >1: normal response
Plt (×10^3^ cells/mm^3^)	271	449	150–450
CD3+ T cells (%; absolute count)	**54.4 (1,134.6)**	**46 (785.2)**	60%–76%
CD4+ T cells (%; absolute count)	45.7 (1,541.2)	**20 (221.2)**	31%–47%
CD8+ T cells (%; absolute count)	**15 (505.8)**	**38 (420.2)**	18%–35%
CD19+ B cells (%; absolute count)	**38 (1,281.5)**	16 (176.9)	13%–27%
CD16+ NK cells (%; absolute count)	3.2 (107.9)	8 (88.4)	3%–15%
CD56+ NK cells (%)	3.7 (124.7)	10 (110.6)	3%–15%
NBT	100	100	>95
CRP	3	**105**	<10
LTT			
PHA	3.5	3.4	≥3
BCG	2.6	**1.3**	≥2.5
Candida	2.5	**1.0**	≥2.5

WBC, white blood cell; Ig, immunoglobulin; Anti-D, anti-diphtheria; Anti-T, anti-tetanus; Plt, platelet; NK, natural killer; CRP, C-reactive protein; LTT, lymphocyte transformation test; PHA, phytohemagglutinin; BCG, Bacillus Calmette–Guérin. The bold values are the abnormal ones.

## Data Availability

All the relevant data of both cases are included in this article. Further enquiries can be directed to the corresponding author.
